# Genome Sequence of *Bacillus butanolivorans* K9^T^ (DSM 18926), an *n*-Butanol-Consuming Bacterium Isolated from Soil

**DOI:** 10.1128/genomeA.01228-15

**Published:** 2015-10-22

**Authors:** Jie-ping Wang, Bo Liu, Guo-hong Liu, De-ju Chen, Rong-feng Xiao, Xue-fang Zheng, Huai Shi, Ci-bin Ge

**Affiliations:** Agricultural Bio-Resources Research Institute, Fujian Academy of Agricultural Sciences, Fuzhou, Fujian, China

## Abstract

*Bacillus butanolivorans* K9^T^ (DSM 18926) is a Gram-positive, spore-forming, strictly aerobic, and *n*-butanol-consuming bacterium. Here, we report the 5.68-Mb genome sequence of *B. butanolivorans* K9^T^, which is the first genomic information of this species that will provide useful information for the genomic taxonomy and phylogenomics of *Bacillus*-like bacteria.

## GENOME ANNOUNCEMENT

The type strain K9^T^ (along with the strains K105, K1012A, and K101) was isolated from soil in Lithuania and was named *Bacillus butanolivorans* sp. nov., due to its ability to tolerate high concentrations of *n*-butanol and to use it as a sole carbon source (1). Its phylogenetically close neighbors are *Bacillus simplex* (2) and *Bacillus muralis* (3) according to their 16S rRNA gene sequence similarity. The best growth of *B. butanolivorans* K9^T^ was achieved at 25°C and pH 7.0 in medium containing 1% (wt/vol) NaCl (1). Notably, there is no other information about *B. butanolivorans* outside its taxonomical description information so far. Given no available genomic information of *B. butanolivorans*, its type strain K9^T^ was selected as one of the research objects in our “genome sequencing project for genomic taxonomy and phylogenomics of *Bacillus*-like bacteria.” Here, we presented the high-quality draft genome sequence of *B. butanolivorans* K9^T^ (DSM 18926).

The genome sequencing of *B. butanolivorans* K9^T^ (DSM 18926) was performed via the Illumina Hieseq 2500 system. Two DNA libraries with insert sizes of 500 and 5,000 bp were constructed and sequenced. After filtering of the 1.22-Gb raw data, the 1.18-Gb clean data was obtained, providing approximately 200-fold coverage. The reads were assembled via the SOAPdenovo software version 1.05 (4), using a key parameter K setting at 77. Through the data assembly, 17 scaffolds with total length 5,682,344 bp were obtained, and the scaffold *N*_50_ was 4,601,026 bp. The average length of the scaffolds was 334,255 bp, and the longest and shortest scaffolds were 4,601,026 bp and 561 bp, respectively. A total of 90.60% clean reads could be aligned back to the genome, which covered 99.72% of the sequence.

The annotation of the genome was performed using the NCBI Prokaryotic Genomes Automatic Annotation Pipeline (PGAAP) (http://www.ncbi.nlm.nih.gov/genome/annotation_prok/) utilizing GeneMark, Glimmer, and tRNAscan-SE tools (5). A total of 5,278 genes were predicted, including 4,994 coding sequences (CDS), 183 pseudo genes, 92 tRNAs, and 8 rRNA genes. There were 3,773 and 2,855 genes assigned to COG and KEGG databases, respectively. The average DNA G+C content was 37.91%, agreeing with the value 37.4 mol% acquired by HPLC determination (1).

### Nucleotide sequence accession numbers.

This whole-genome shotgun project has been deposited at DDBJ/EMBL/GenBank under the accession no. LGYA00000000. The version described in this paper is version LGYA01000000.
